# Fatigue Behavior of Additively Manufactured Stainless Steel 316L

**DOI:** 10.3390/ma16010065

**Published:** 2022-12-21

**Authors:** Andrea Avanzini

**Affiliations:** Department of Industrial and Mechanical Engineering, University of Brescia, 25128 Brescia, Italy; andrea.avanzini@unibs.it; Tel.: +39-0303715526

**Keywords:** additive manufacturing, fatigue, stainless steels, 316L

## Abstract

316L stainless steel is the material of choice for several critical applications in which a combination of mechanical strength and resistance to corrosion is required, as in the biomedical field. Additive Manufacturing (AM) technologies can pave the way to new design solutions, but microstructure, defect types, and surface characteristics are substantially different in comparison to traditional processing routes, making the assessment of the long-term durability of AM materials and components a crucial aspect. In this paper a thorough review is presented of the relatively large body of recent literature devoted to investigations on fatigue of AM 316L, focusing on the comparison between different AM technologies and conventional processes and on the influence of processing and post-processing aspects in terms of fatigue strength and lifetime. Overall fatigue data are quite scattered, but the dependency of fatigue performances on surface finish, building orientation, and type of heat treatment can be clearly appreciated, as well as the influence of different printing processes. A critical discussion on the different testing approaches presented in the literature is also provided, highlighting the need for shared experimental test protocols and data presentation in order to better understand the complex correlations between fatigue behavior and processing parameters.

## 1. Introduction

Stainless Steel (SS) 316L is widely used in several industrial sectors thanks to a favorable combination of high corrosion resistance, good mechanical properties, weldability, and formability. Typical applications include body implants and heat exchangers [1], but this grade of stainless steel has also been successfully used in a range of environments such as in the chemical, petrochemical, nuclear, and food industries [2]. 

In its powder form, 316L is also very appropriate for Additive Manufacturing (AM), and out of the different metals suitable for AM, 316L is one of the most commonly used because of its low thermal conductivity and high melting point, combined with limited sensitivity to the presence of oxygen and high absorptivity in infrared [3]. AM actually encompasses a large number of technologies, whose working principles and manufacturing steps may vary substantially and it is well recognized that AM metals exhibit a peculiar microstructure, which is significantly different when compared with nominally equivalent alloys obtained with traditional routes [4,5]. In particular, while a fully austenitic phase is generally present when processing with conventional manufacturing methods, for AM 316L the presence of δ-ferritic phase, oxides, or σ-phase has been reported, depending on the process [6]. AM parts may also exhibit significant porosity and higher surface roughness in comparison with fully dense material from traditional routes, which may affect static and fatigue properties. As a consequence, specific material characterization is needed for each process type and, in the past, a large number of investigations has been published, concerning both the relationship between processing and microstructure, both the resulting mechanical properties in comparison with traditional counterparts (see for example review papers [7,8,9,10,11]). For AM 316L most of the current literature deals with more mature processes, like Powder Bed Fusion (PBF), and, to a lesser extent, Directed Energy Deposition (DED). However, an increasing amount of research is also being published for other emerging technologies such as Binder Jetting (BJ) and metal extrusion (ME), in particular Fused Filament Fabrication (FFF). Overall, the current literature shows that AM 316L can match, or even exceed, tensile properties of conventionally manufactured counterparts, but data still present some variability, depending on the printing method and on the processing parameters. PBF and DED allow the production of dense parts with higher Yield Strength (YS) and Ultimate Tensile Strength (UTS) than BJ and FFF, whereas sintering-based technologies seem to exhibit somewhat higher strain at failure (ε_R_) [3,12,13,14,15].

Of course, since 316L is often used for structural applications where cyclic loads may be expected, it is paramount to gain some knowledge of fatigue behavior, especially because the fatigue mechanisms of additively manufactured materials and parts are not yet completely understood [16]. Several factors may affect the fatigue response of AM metals, including processing parameters, building orientation, presence of porosity or defects, surface finish, and heat treatments [17,18,19]. Published studies often consider different combinations of these factors and, depending on the purpose of the investigations, different fatigue regimes (i.e., low or high cycle number), cycle types (i.e., alternating or pulsating), and stress states (i.e., uniaxial or multiaxial) may be of interest. Consequently, while a considerable number of publications are already available in the literature for fatigue of AM 316L, it can prove surprisingly difficult to compare the available results. In the present work, a thorough overview of the current literature on fatigue of AM 316L is presented and available data are analyzed as a function of key factors, such as AM process type, surface finish, build orientation, and heat treatment. Given the strong link between fatigue behavior and surface or microstructural features resulting from the processing route, a concise summary of additive manufacturing technologies applied to 316L and the resulting microstructure is first presented. Then, a presentation and critical discussion of available fatigue data follow.

## 2. Additive Manufacturing Technologies for Stainless Steel 316L

Many technologies are currently available for metal 3D printing and while different classifications are possible, for the purpose of the present review they will be broadly divided into Powder Bed Fusion (PBF), Directed Energy Deposition (DED), and Binder Jetting (BJ), with the addition of Fused Filament Fabrication (FFF), an extrusion-based method which has recently been employed for 316L.

In PBF an energy source is used to melt the metal, layer by layer, in a bed of powder. Each time a layer is melted or sintered, another layer of powder is deposited on the bed and the platform is lowered to melt it. This technique needs gas to avoid the oxidation of metal, such as argon or nitrogen, or a vacuum. This type of 3D printing can use a laser or an electron beam to melt the metal. For 316L, laser powder bed fusion (L-PBF) is by far the most common process and a large body of the current literature deals with Selective Laser Melting (SLM) or sintering, such as EOS© Direct Metal Laser Sintering (DMLS). Comprehensive reviews concerning the influence of processing parameters on achievable density, type of defects, and microstructure in relation to mechanical properties for L-PBF 316L can be found in [1,13,14,20,21,22].

In DED techniques the melting and deposition of the material occur at the same time, usually, through the use of a laser [23], in which case the process can be denoted as Directed Laser Deposition (DLD or L-DED). The deposition of the fused material is on a platform, which can be moved on some axes and the technique also needs an inert gas to avoid oxidation. For 316L, the use of this technique has been reported in [3,24,25], whereas a review of resulting microstructures and mechanical properties can be found in [12]. While the material melted by the laser is most often in powder form (L-DED-P), a wire form can also be used (L-DED-W) which offers a higher efficiency of material usage and higher deposition rates [26]. Laser-engineered net shaping (LENS) has also been documented for 316L in [27]. Another DED manufacturing approach is represented by Wire Arc Additive Manufacturing (WAAM), which uses an electric arc to melt metallic wire feedstock instead of a laser beam as a fusion source. For 316L, the use of this technology was reported in [28,29]. The Binder Jetting technique [30] also relies on the use of a powder bed but with a different working principle. In fact, BJ is a multistep process in which a binder is spread over a layer to glue the powder in and between successive layers. After curing, the final part is obtained via a sequence of de-powdering, sintering, infiltration, annealing, and finishing [10]. For 316L, recent investigations were reported in [6,31,32,33]. In general, unlike PBF technologies, binder jetting is compatible with virtually any powdered material and is attributed a real potential to surpass L-PBF thanks to the widest selection of materials of all AM processes [34], yet as-printed parts show lower relative density, higher surface roughness and lower resolution in comparison with L-PBF. Finally, an alternative to the widespread beam-based techniques is provided by filament extrusion-based additive manufacturing of metals (FFF), in which a metal powder is mixed with a polymeric binder to form a filament. After filament deposition on a platform to allow shaping, the binder is removed in a debinding step, and the remaining material is sintered, similarly to BJ, with the advantage of low cost, anisotropy, and low health hazard. The use of FFF for 316L has been recently reported in [15,35,36].

## 3. Fatigue Studies on AM 316L

In the past, fatigue properties of AM 316L were investigated by several groups but the published literature may vary significantly in terms of the aim of the research, testing protocols, and the way results are presented. For the purpose of the present review, the available literature is first categorized according to the AM technology (i.e., PBF, DED, BJ, and FFF) and then considering the main process-related factors. In the context of the literature concerning fatigue, reported data mainly refer to combinations of the following factors:

- Printing parameters: i.e., Laser Energy Density (E_d_) or Layer Thickness (t)
- Build orientation: Horizontal (XY), Vertical (Z), Diagonal (45°)
- Surface Finish: As-Built (AB), Machined (M), Polished (P), or Surface Treatment (ST)
- Heat Treatment: No treatment (N), Stress-Relief (SR), Annealing (ANN), Hot Isostatic Pressing (HIP), Precipitation Hardening (PH).

Further differences between the available literature data may exist when considering test protocols. Fatigue tests on AM 316L have been conducted by changing the following conditions:

- Loading mode: Axial (AX), Rotating Bending (RB), Reversed Bending (RevB), Torsional (T), Multiaxial (M-AX)
- Specimen type and geometry: Cylindrical (C), Flat (F)-Dogbone (DB), Hourglass (HG)
- Test control variable: Load (Stress-based), Strain (Strain-based)
- Fatigue Load Ratio: R (Alternating R = −1, Pulsating R ≥ 0)

When categorizing fatigue studies, papers can be further classified depending on the test method, with a distinction between the Stress-Based and Strain-Based approaches. The former is the most common and represents high-cycle applications adequately, but it is less accurate for low-cycle applications. The Strain-Based approach is less frequently used but more accurate for low-cycle fatigue testing [37]. As a consequence, this distinction is also almost equivalent to a categorization based on the fatigue life cycle number, i.e., between High Cycle Fatigue (HCF) and Low Cycle Fatigue (LCF). Considering AM316L, most literature works adopted a Stress-based approach and the life range between 10^6^ and 10^8^ cycles is the most investigated. A few recent investigations considered the extension to Very High Cycle Fatigue (VHCF), but the literature is even scarcer [38,39]. Consequently, if not otherwise stated, fatigue results discussed in this review mainly refer to HCF. Strain-based approaches are less frequent and usually they cover LCF, although life may range between 10^2^ and 10^6^ cycles. Given the limited number of references available for AM316L, these are discussed in a separate paragraph of the paper.

In some studies, stepped test protocols (STP) for accelerated fatigue testing were also used and a few studies addressed aspects related to Fatigue Crack Propagation (FCP) on compact tension (CT) specimens, the role of artificial defects (AD), or residual stresses. A large body of existing literature deals with L-PBF technology, which is certainly the most mature, with the first paper reporting fatigue data on SLM-316 [40] dating back to 2013. A summary of the studies on L-PBF included in this review is provided in Table 1 for Stress-based and Table 2 for Strain-based approaches, respectively.

More recently, papers concerned with DED, BJ, and FFF were also published, as listed in Table 3. As apparent by the limited number of papers, the knowledge of fatigue behavior for these technologies is still limited to a few preliminary studies.

Given the variety of test protocols employed, a direct comparison between different studies is not straightforward, also because raw data are often not available and fatigue curves are presented only in graphic form. In order to overcome this difficulty, in the present review the software WebPlotDigitizer 4.6 © [80], which allows manual or automatic conversion of digital plots into tabulated data, was used for the digitalization of life curves from different papers. Unfortunately, there is also a lack of uniformity in the way fatigue life curves are reported, with different authors using, for example, alternating stress σ_a_, maximum stress σ_max_, or stress range Δσ. A further complication is that different load ratio R (i.e., the ratio between minimum stress σ_min_ and maximum stress σ_max_ in a load cycle) may be employed. In order to allow direct comparison between different studies, following [81], after computation for each dataset of mean and alternating stresses in the cycle, all data were corrected for mean stress employing the Smith-Watson-Topper (SWT) correction, obtaining an equivalent alternating stress σ_ar_, as per Equation (1):

(1) $$\sigma_{ar}=\sigma_{max}\sqrt{\frac{1-R}{2}}$$

While other equivalent alternating stress can be defined, i.e., based on Walker or Goodman approaches or their modifications [82], SWT correction has the practical advantage that relevant data (σ*_max_* and *R*) can easily be retrieved from the papers, without the need to determine additional parameters. In the following paragraphs, therefore, all life curves will be presented in the form of σ_ar_ -N_f_ data, where N_f_ is the number of cycles to failure. The interested reader is redirected to the referenced studies to retrieve the actual data in the form they were originally published.

## 4. Fatigue of L-PBF 316L

Fatigue is affected by microstructural aspects such as phase composition, grain size, and achievable density [83], and since L-PBF is the most commonly used technology for AM 316L, a large body of literature is concerned with investigations on microstructural features, defects, porosity, and mechanical properties. 

316L stainless steel is in a chemical composition range where solidification can occur either with a primary δ-ferritic phase or with a primary austenitic γ-phase. Depending on the process type, tuning of processing parameters, and heat treatment, AM 316L parts may exhibit full γ-austenite or include δ-ferrite, in contrast to the fully austenitic phase when processed by conventional manufacturing methods [6]. In L-PBF, the observed microstructure of 316L is generally fully austenitic with no indication of any solid-state phase transformation [8,69,84,85]. As remarked in [8], the microstructure of AM-produced steels is dominated by the solidification and solid-state phase transformations. Its features are smaller than those in the conventionally produced counterparts, usually at least by an order of magnitude, because of faster heat-up and rapid solidification. In line with this interpretation, the grains observed in L-PBF are finer than the ones in conventional processes [22,69,85], and in one austenite grain, many tens or hundreds of solidification cells may be present [8]. Usually, a strong crystallographic texture with the <001> direction aligned along the build direction (i.e., against the direction of fastest heat removal) is observed [86], but depending on the scanning strategy such a strong crystallographic texture is sometimes not present, or can be changed by tuning laser power and hatch spacing [87]. Considering the influence of microstructure on fatigue, austenitic stainless steels in their wrought form are known to be more susceptible to Fatigue Crack Initiation (FCI) at annealing twin boundaries and high angle grain boundaries (HAGB) than nano/micro-scale defects such as inter-metallic inclusions and voids [88]. When considering L-PBF, as discussed in [18] for a L-PBF 304 stainless steel, by leveraging the relationship between local thermal input and the resulting microstructure/properties it may be possible to tailor microstructure to meet specific loading requirements. For example, in areas where high strength and resistance to FCI are required, high cooling rates may be beneficial to produce finer microstructures, whereas larger grains are beneficial for areas where crack growth is critical, and a reduction in the cooling rate can lead to a coarsening of the microstructure. However, for L-PBF 316L such fine-tuning of processing parameters for improvement of fatigue resistance has not been reported yet. The use of different processing parameters not only results in different microstructures but also affects porosity levels as well as the size, position, and shape of defects. In turn, these may affect fatigue by acting as stress concentrators, potentially hindering the possibility of producing reliable, fatigue-resistant additive-manufactured (AM) parts. Considering porosity, L-PBF 316L is one of the materials where there has been significant success in achieving near full density, as remarked in [14] in which several works on L-PBF parameters were considered. Most studies consider laser energy density (E_d_) as the main parameter to optimize part densification, although different combinations of laser parameters can be used to optimize E_d_. For higher densification (above 99%), the optimal energy density values are scattered in the range between 50 J/mm^3^ and 150 J/mm^3^. Although several works on fatigue surprisingly omit this essential information, and different methods can be used to assess density, the published literature is quite consistent in reporting density values in the range of 99.3–99.9% [44,54,57,89]. 

Given the relatively high number of fatigue studies, for L-PBF 316L comparative analyses of fatigue data are possible, although, as apparent from Table 1, several different combinations of processing and testing factors were used. In the following paragraphs, fatigue data from the literature will be compared considering separately the influence of the main ones and taking as a reference the data for AB condition, i.e., no surface machining or treatment and no heat treatments.

### 4.1. Fatigue of L-PBF 316L in AB Conditions

**Figure 1:**
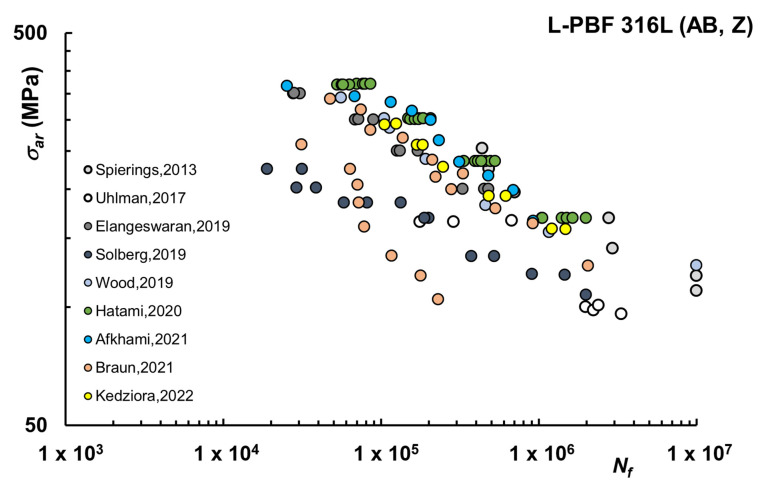
Fatigue life curves L-PBF 316L–As-Built (AB), Vertical (Z) samples [40,43,48,50,51,55,57,58,66].

Fatigue resistance of specimens or mechanical components is deeply influenced by surface finish and is well known for conventionally manufactured steels, and specimens for fatigue testing should be carefully prepared by machining and/or polishing, as per international standards [90,91]. On the other hand, ideally, AM components should be designed for use in their net shape without machining operations, otherwise, a key advantage of AM would be lost. As a result of these contrasting needs, AM studies on fatigue are often carried out considering AB (or as-fabricated) conditions, eventually in comparison with machining. A summary of fatigue data for AM 316L in AB condition is presented in Figure 1, in which results refer to the most common condition of vertical printing direction (Z) for Stress-based approaches [40,43,48,50,51,55,57,58,66]. The surface roughness was not always measured, and papers may report alternatively R_a_ or R_z_, or even S_a_. In general, values are within an approximate range of 4–21 μm for R_a_ and 24.5–132 μm for R_z_. Similarly, porosity level or density is declared only in three studies [40,48,57], with density values in the range of 99.3–99.9%, in line with the expected nearly full density of L-PBF steels.

Overall, mainly as a consequence of high roughness and the presence of pores, the results show that the fatigue properties for AB condition are lower than conventional (machined) counterparts. Run-out at 10^7^ cycles for σ_ar_ around 120 MPa [40,51] can be noticed, whereas for wrought 316L run-out at 10^7^ with σ_ar_ up to the range between 275 and about 330 MPa were reported [42,51]. Of course, such a comparison has more sense when machined or polished AM 316L is considered since this is the standard testing condition for forged or cast materials, in which case fatigue test data are always available only for machined samples. However, one of the advantages of AM technologies is the possibility of obtaining near-net shape components with complex geometries, such as those from topology optimization, without further machining. The data available therefore shows that some improvement for current technologies is still needed to make as-fabricated AM 316L competitive with its traditional counterparts.

The high scatter between different studies is essentially a consequence of the use of different commercial systems in which powder feedstocks, processing conditions (i.e., atmosphere, platform temperature), or printing parameters may vary significantly. Moreover, since details of commercial printing machine set-up are often covered by patents, values of relevant parameters, such as hatch distance, scanning strategy, or even laser power, are overlooked or simply not reported in fatigue studies. When considering the available literature, the single parameter most commonly used to represent processing is the laser energy density E_d_ which is defined as per Equation (2):

(2) $$E_{d}=\frac{P}{vht}$$

where *P* is the total energy emitted by the laser in unit time, the scanning speed (*v*) is the velocity of the laser spot, hatch spacing (*h*) is the distance between adjacent scan vectors, and layer thickness (*t*) is the depth of powder layer melted on the powder bed [14]. For the studies considered in Figure 1, E_d_ is in the range of 26.7–60 J/mm^3^. Notably, among examined works, the lowest fatigue lives were those reported in [50], in which case the lowest value of E_d_ was used and X-ray Computed Tomography (XCT) displayed unfavorable orientation of large defects and in [43], where the highest roughness values were present. In [58] for the series of specimens tested with R = −1, the fatigue strength of as-built materials was also significantly reduced by the surface roughness, tensile residual stresses, and process-induced defects. The influence of layer thickness was specifically considered in [55], showing that increasing the layer thickness from 30 to 50 μm has a minor negative impact on fatigue strength.

### 4.2. Fatigue of L-PBF 316L: Influence of Surface Finish and Treatment

**Figure 2:**
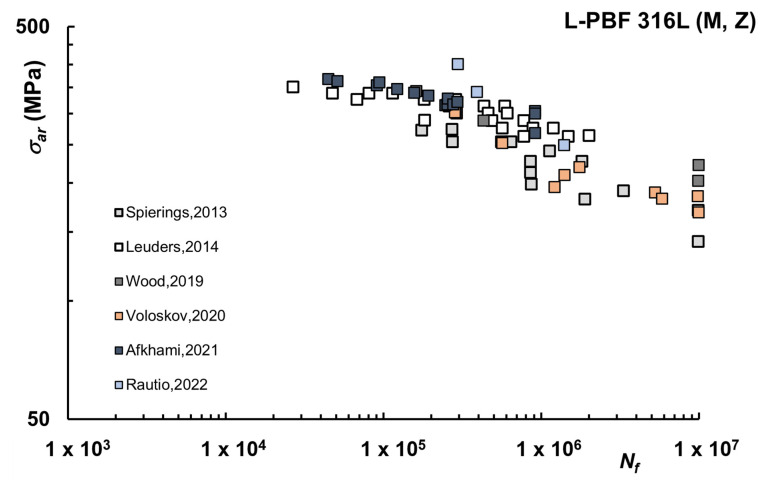
Fatigue life curves L-PBF 316L–Machined (M), Vertical (Z) samples [38,40,41,51,57,67].

Ideally, fatigue tests should be carried out using specimens with a perfectly smooth surface, since the fatigue limit determined under this ideal condition can eventually be reduced, as a function of the roughness achievable on the final surface state, by means of knock-down factors, often available in textbooks for conventionally manufactured materials [92]. This approach has been adopted by several researchers also for AM materials, but unfortunately, the way surface finishing is carried out can be quite different depending on the study. The most common finishing conditions are turned, ground, and polished, though some studies simply refer to machined conditions. Fatigue life curves for L-PBF 316L, again considering only samples not subjected to heat treatment, are reported in Figure 2 for the machined or turned condition in which case R_a_ is in the range of 0.6–1.8 μm and R_z_ is 3.7–11 μm, although this essential information is not always reported [38,40,41,51,57,67].

**Figure 3:**
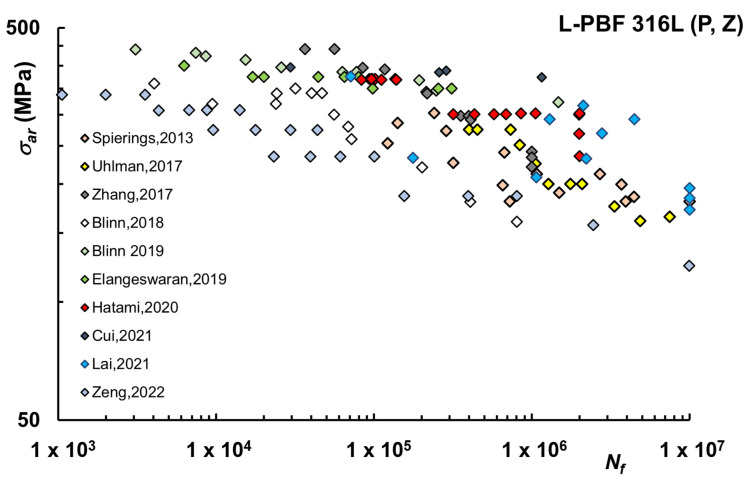
Fatigue life curves L-PBF 316L–Polished (P), Vertical (Z) samples [1,40,43,46,47,48,55,61,65,74].

Figure 3 refers instead to ground and polished (either manually or after preliminary turning) samples, in which case, R_a_ is in the range of 0.05–1.08 μm and R_z_ is 0.4–4.96 μm [1,40,43,46,47,48,55,61]. For both machined and polished samples, the curves refer to vertical (Z) printing orientation. 

As observed, the comparison with AB is generally favorable because the roughness of AB specimens is relatively high. In terms of run-out at 10^7^ cycles, the values of the corresponding σ_ar_ are in higher ranges (about 140–220 MPa for machined and 120–190 MPa for polished), although lower than conventionally machined.

**Figure 4:**
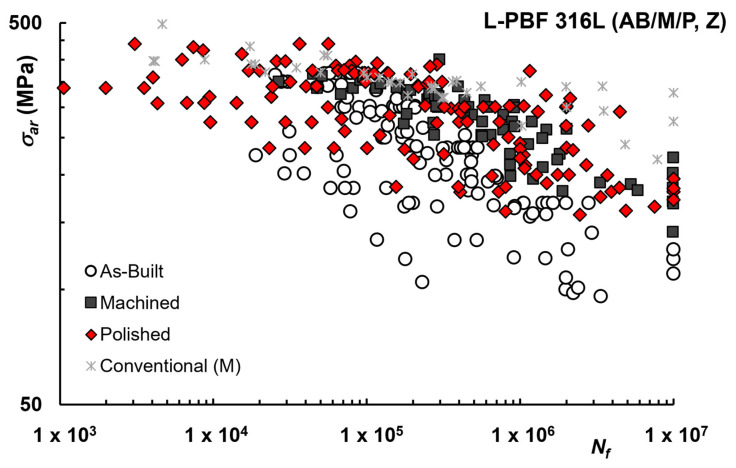
Fatigue life curves L-PBF 316L–Comparison of AM AB, Machined and Polished and Conventional 316L.

Finally, in Figure 4, an overall comparison of datasets for AB, Machined, and Polished is reported to better appreciate the effect of each condition, including data for conventional 316L gathered from different sources [2,42,46,51,93]. 

The positive influence of surface machining can be more clearly noticed, although unfortunately, the current amount of data available for AM metal alloys do not allow reliable determination of knock-down factors.

Notably, the comparison between polished and machined samples shows similar results, although a significant scatter of the data can be observed, even for the polished ones.

The comparison with conventional 316L appears to be more favorable for lower cycles numbers, in which case data for AM machined or polished are well overlapping, while for higher cycle numbers, despite some dispersion of the data for conventional material, a significant difference remains, with a steeper reduction of fatigue strength for AM condition.

For AM 316L a significant scatter is seen. While not all processing parameters are disclosed in the analyzed literature, such scatter could be related to the fact that for AM materials the properties are highly process-dependent and the number of processing parameters is potentially huge. On the other hand, another possible explanation is that in some cases samples are turned or machined from bulk material, originally printed in the form of cylindrical or rectangular blocks, while in other studies they are obtained from near net shape samples, by peeling off a thin layer of material.

As discussed in the literature, porosities may be present in the subsurface layers of AM samples [94,95], and studies have shown that defects closer to the surface can be more detrimental to fatigue resistance [16,96]. These can be completely removed (or not) by milling or polishing, depending on how deep the surface is machined, with respect to defect size and position. Moreover, according to [61], the machining process can partially release the internal residual stress due to material removal, therefore the machined and polished samples may even have higher fatigue strength compared to the polished-only samples.

**Figure 5:**
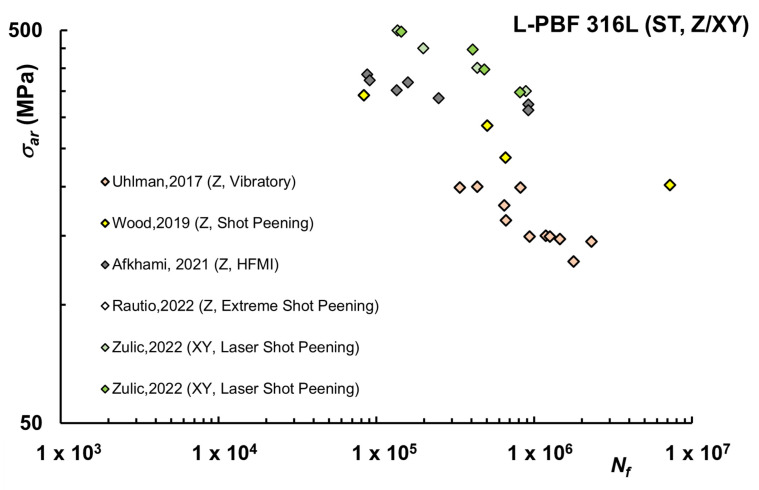
Fatigue life curves L-PBF 316L after surface treatment on Vertical (Z) or Horizontal (XY) samples [43,51,57,67,68].

An alternative to machining or polishing operations is represented by surface treatments, which may alter roughness, usually by mechanically reducing heights of peaks and valleys of the profile and/or inducing a compressive stress state. For AM 316L, different approaches were reported in the literature, including Shot Peening (SP) [51,67], Laser Shot Peening (LSP) [68], High-Frequency Mechanical Impact (HFMI) [57], and vibratory finishing process (VF) [43]. A summary of the life curves under these conditions is reported in Figure 5. 

Overall surface treatments based on shot peening seem to improve fatigue resistance at levels comparable with machined surfaces. One of the investigations [67] reported extremely high values for applied cyclic stress, with fatigue strength in the LCF regime even exceeding the static strength of the AB material, which could be attributed to the extremely high level of compressive stress induced by severe SP. HFMI seems instead associated with a limited advantage in comparison with other techniques.

### 4.3. Fatigue of L-PBF 316L: Influence of Build Orientation

**Figure 6:**
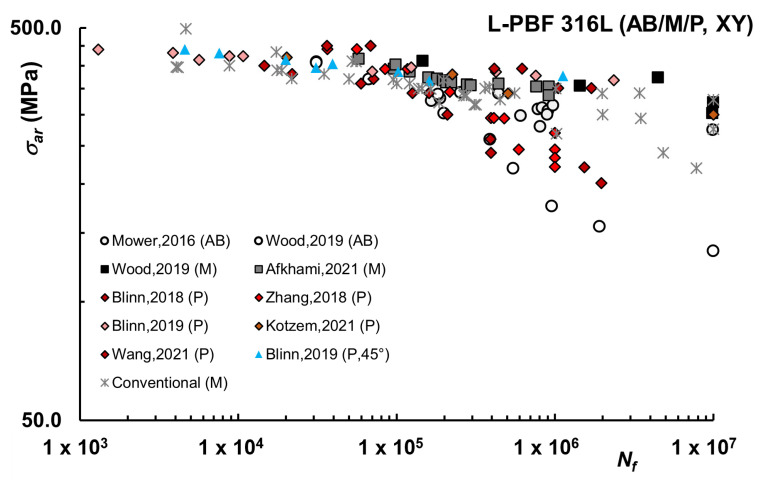
Fatigue life curves L-PBF 316L–Horizontal (XY) samples [42,44,46,47,51,57,60,62].

A summary of the available data for L-PBF 316L printed horizontally (XY) is reported in Figure 6, including both AB and M or P surface conditions. Ideally, direct comparisons of orientation should be made within a single study, as samples from different studies may exhibit inter-build variations, such as different powder chemistries, software settings, machine model and year, and build geometries. Nevertheless, the data reveals that the fatigue strength of samples flat printed is higher, the slope in the finite life regime is less steep and that at run-out, for most favorable conditions, σ_ar_ can be equal to or even higher than conventional material.

In general, the build direction of the parts fabricated by PBF processes has a significant impact on fatigue properties [96] since multiple orientation-related aspects may influence fatigue behavior, as reviewed in [16]. First of all, PBF parts are built layer-by-layer and for AB conditions this may result in different roughness depending on direction. Moreover, irregularities on the surface of vertical samples may act as notches with potentially sharp radii, oriented perpendicular to the loading direction in vertical samples, which provides a preferential crack initiation site. Building orientation also determines the size, distribution, and direction of the processing defects. In SLM steels lack fusions and porosities are typically elongated perpendicular to the building direction [16] and horizontally printed samples possess a higher distribution of porosities with smaller diameters. The combination of the bigger size of the defects and their alignment normal to the loading direction may explain inferior fatigue properties often reported in vertical samples in comparison to horizontal ones. Another feature that is different for the specimens with dissimilar building orientations is the arrangement of interfacial weak links between layers, which can be more unfavorable for vertical specimens loaded perpendicularly to the plane on the layers. In addition, the presence of planar defects between the layer interface may maximize stress concentrations.

Overall, numerous studies showed that fatigue strengths and limits of SLM steels improved by manufacturing them in the horizontal direction and fatigue results analyzed in the present review seem to confirm this trend for L-PBF 316L, both in AB and M or P condition. In particular, in [50] higher values for the roughness of vertically built specimens were reported (AB condition), and correspondingly higher fatigue strength for XY build directions in comparison with Z. Similarly, in [41] DMLS 316L specimens built vertically produced fatigue strengths at lifetimes of 10^5^–10^6^ cycles of only about 30% of that generated by horizontally-built DMLS material, as also reported in [67], in which case data refer to stress-relieved condition. Investigations on machined or polished samples were reported in [45,46,59,81], again showing improved fatigue properties for the horizontal samples.

Of course, Figure 6 also shows that unfavorable combinations of processing parameters, such as layer thickness or low power, may also result in low fatigue properties for horizontally printed samples, as clear from a series of studies by Zhang [45,82]. As a side note, only one study is available for diagonal build direction [48], which interestingly shows fatigue properties similar to XY.

Finally, it should be noted that fatigue data shown in Figure 6 for conventional 316L cumulative refers to different studies [2,42,46,51,93]. Notably, only in a few works [46,51,64] was the conventional material was tested under the same conditions as AM 316L under investigation. More frequently, the comparison is indirect, assuming as a reference literature data, especially those reported in [2] for wrought material. However, fatigue data reported for the conventional processing route may also exhibit some variability, depending on the reference chosen as well as on the processing route considered, which may include Continuous Casting, Powder Metallurgy, or Metal Injection Moulding [46,78,97].

### 4.4. Fatigue of L-PBF 316L: Influence of Heat Treatment

Among the diverse types of heat treatments reported in the literature for AM 316L, the most common is Stress Relief (SR), which is often performed on AM products and samples to release tension in the material at the end of the printing process. For SR, the temperature is relatively low and no changes in the microstructure occur, but maximum temperatures and durations varied quite significantly among the studies, ranging between 350–650 °C [46,70] and 1–9 h [68,98]. The other possible treatments include Annealing (ANN) and Hot Isostatic Pressing (HIP), which involves a combination of high temperature and pressure. For ANN HT, higher temperatures than SR are used (900–1090 °C) with durations between 10 min and 2 h, eventually followed by gas quenching in the furnace [49,61,82,99]. The HIP is conducted at temperatures between 1150 °C and 1190 °C, with applied pressure between 1000 and 1450 bar and a duration of 3–4 h [41,42,54,100]. In [77], some HIPed samples were subjected to Precipitation Hardening (PH) with water cooling to reduce the sigma phase formation phenomenon.

Interpretation of results from studies concerning the influence of HTs can be difficult because different combinations of surface conditions and printing direction are typically considered. Since both factors alter significantly fatigue response, they may hinder the influence of HT and lead to different conclusions depending on the starting point. For these reasons, in this review, fatigue data for HT condition are presented separately for AB and machined or polished surfaces, including first the only samples printed vertically and discussing separately data from studies on horizontally printed samples. The data are reported cumulatively for the different HTs, not considering slight differences in thermal histories, and the interested reader may refer to references in Table 1 for specific datasets.

**Figure 7:**
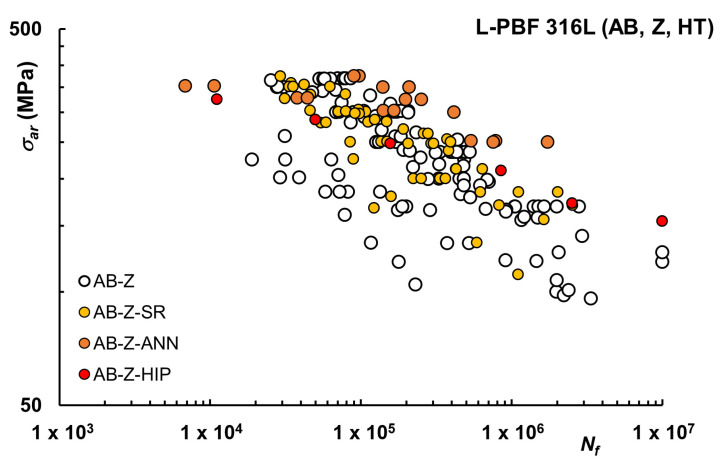
Fatigue life curves L-PBF 316L after HT, As-Built (AB), vertical (Z) samples.

In Figure 7 data available for AB conditions after SR, ANN, or HIP are shown for vertically printed samples. SR increases fatigue resistance when starting from AB condition. This improvement could suggest that SR, as the name implies, may actually reduce residual stresses leading to a more favorable condition for fatigue. However, it should be noted that in [48] it was instead reported that the removal of residual stresses via an SR heat treatment does not significantly influence the fatigue behavior, with similar results for AB- and SR-treated samples. For AB, ANN seems to produce fatigue data in a higher stress range than SR, but data for this condition on vertically printed samples are limited to ref. [49]. HIP does not seem to be particularly effective in improving fatigue behavior for AB condition, in which case the expected benefit of reduced porosity for AB samples is hindered by the detrimental effect of surface roughness, but also in this case only one reference is available [42]. Interestingly, in [54] sub-grained cellular microstructures were observed for AB and SR conditions, and coarse-grained microstructures for fully annealed and HIP samples, concluding that AB condition can be preferred when higher strengths and cyclic loading at higher stresses are required. When higher ductility and lower cyclic stresses are needed, a fully annealed state can be preferred.

**Figure 8:**
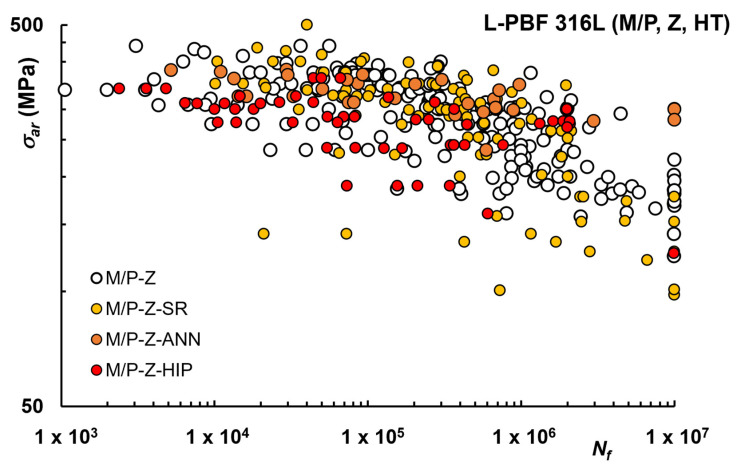
Fatigue life curves L-PBF 316L after HT, Machined (M) or Polished (P) condition, Z samples.

In general, investigations in the context of fatigue studies for AB conditions are limited in number, whereas more papers are available for machined or polished conditions, as reported in Figure 8.

When considering M or P surfaces (see Figure 8), the influence of SR seems limited, with higher overlapping of the data. This could indicate that after machining the surface, the relevance of SR in reducing the residual stress could be diminished, possibly because the machining process can partially release the internal residual stress due to material removal. Unfortunately, the number of studies in which residual stresses were measured is limited, and further investigations would be useful.

The results presented in the literature after ANN HT, for machined or polished condition, are instead not conclusive. In [65], the effect of typical AM alloy cellular structure in as-fabricated condition on the fatigue behavior was investigated, especially considering dislocation-based deformation mechanisms, showing that after annealing at 1050° for 10 min and quenching, fatigue properties were lower than in AB state. This was attributed to the absence of such cells after HT, resulting in different dislocation slip modes, although it should be noted that fatigue data were limited to four samples for each condition. On the contrary, in [61], after HT at 900 °C for 2 h an improvement of fatigue properties was reported, as a consequence of the effective removal of residual stresses. Considering the data in Figure 8, no clear improvement after ANN HT on M/P samples can be noticed.

HIP does not seem to be particularly effective in improving fatigue behavior and since data are similar to ANN, which is a simpler post-processing route, this latter could be preferable. According to [69], a HIP process is only recommended under low-level HCF load since UTS and the resulting performance in ranges of higher load levels is significantly reduced through the temperature–time profile of this kind of post-treatment. A similar conclusion, reported in [54], is that when higher ductility and performance in a very high cycle fatigue regime is desired, coarse-grained microstructures from full annealing or HIP can be preferred. Very recently, in [74] HIP heat treatment improved the fatigue life of specimens at high stresses (σ_max_ > 250 MPa), while the As-built state was very similar for lower stress applications.

**Figure 9:**
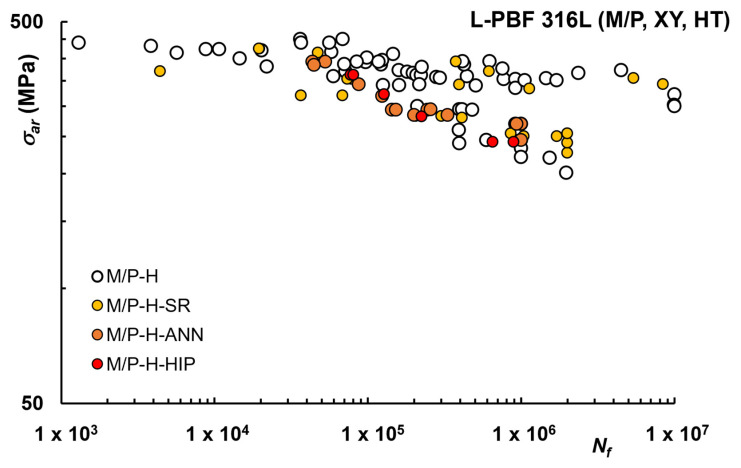
Fatigue life curves L-PBF 316L after HT, Machined (M) or Polished (P) condition, XY samples.

Finally, in Figure 9, the effects of various HTs on horizontally printed (XY) samples are reported, considering only M/P specimens.

Additionally, in this case, HTs do not seem effective in improving fatigue. For horizontal samples in [82], degraded fatigue resistance after ANN HT was reported, with crack initiation occurring at thermally-induced defects, as the microstructural defects are removed by recrystallization and grain regrowth.

## 5. Fatigue of L-DED 316L

DED includes a sequence of physical phenomena, such as rapid heating, melting, potential vaporization, and rapid cooling, and to date, the stability of the microstructure of components produced via DED, which takes place under non-equilibrium conditions, is poorly understood. According to [12], after the solidification of metallic parts produced by DED, not only columnar grains, which represent an elongated morphology that grows in the direction of a maximum thermal gradient, but also columnar-equiaxed grains, and equiaxed grains are the structure morphologies that can be formed as a consequence of various cooling rates and thermal gradient values. Interestingly, in [101], Electron Backscatter Diffraction (EBSD) results of deposited 316L revealed random grain boundaries without significant texture. Depending on building orientation, the microstructure has also been reported to be either more homogeneous or consisting of large dendritic grains [25]. A peculiar aspect is that because of the complex heat transfer during the process different structures can be found depending on the height along the building direction. Due to different cooling rates, finer microstructure and higher microhardness are expected for the bottom and top of the DED components, which undergo higher cooling rates with respect to other areas. In L-DED processing of 316L, it has been observed that in the regions along the borders of the solidification cells the micro-segregation during solidification may lead to the formation of fine ferritic films up to 5–10% [25,27,102,103]. After heat treatment, the austenitic phase remains dominant, but a reduction in the ferrite phase with increasing heat-treatment temperature was noticed [98]. The few investigations on L-DED-W and WAAM also showed that the microstructure consists of two phases, with about 4–5% δ-ferrite [20]. L-DED is a class of laser-based additive manufacturing processes considered capable to synthesize full-density high-performance metallic components. For 316L density values up to 99.9% were reported in [26,46].

**Figure 10:**
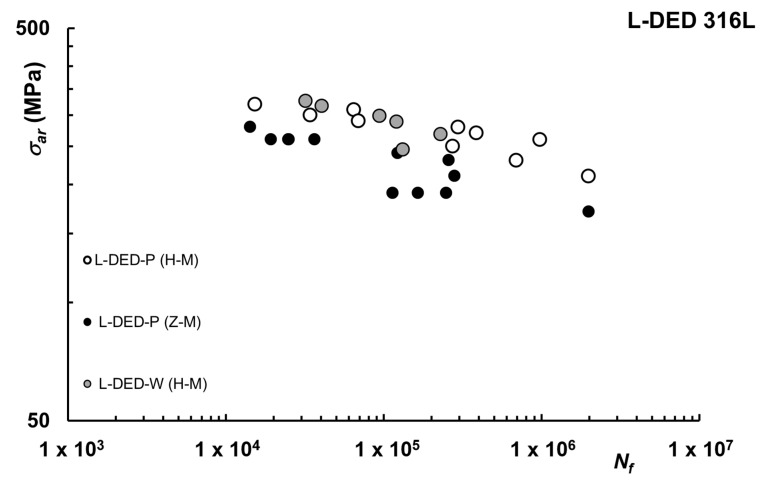
Fatigue life curves L-DED 316L [26].

Considering the fatigue of L-DED 316L, current literature is scarce, with only a few papers from the same research group available, as shown in Figure 10.

In [46], L-DED-P was used to print cuboids which were afterward turned to their final geometrical shape to obtain specimens with the axis aligned either vertically or horizontally. Under constant amplitude fatigue tests, the AM specimens showed a decreased fatigue strength compared to the CC specimens, which was mainly caused by defects in the AM materials, in particular oxide inclusion. Anisotropic fatigue behavior with regard to the building direction was reported, with higher fatigue strength, lower scatter, and flatter slope for horizontally built specimens. In [23], L-DED-P and L-DED-W were compared, considering only horizontal direction. For both configurations, crack initiation occurred at process-induced nonmetallic inclusions which strongly influence the fatigue lifetime. L-DED-W showed a lower fatigue limit at 2 × 10^6^ cycles, caused by substantially bigger inclusion sizes, suggesting that to improve fatigue strength the size and number of nonmetallic inclusions have to be minimized by optimization of the oxidation prevention strategy.

## 6. Fatigue of BJ and FFF 316L

For BJ technologies, in [31], the presence of both austenitic and ferritic phases was reported, with detrimental iron carbides at the grain boundaries and oxides, depending on sintering conditions. The presence of the δ-ferrite phase is reported also in [56], and according to [32], high sintering temperature introduces a higher level of the δ-ferrite phase. One of the major drawbacks when considering BJ is the difficulty in reaching full-density parts after the post-sintering, leading to reduced mechanical performance. While porosity is also affected by part orientation, layer thickness, and other processing parameters, BJ parts usually exhibit higher porosity when compared with PBF or DED methods. Density evolution during sintering depends on sintering temperature (the higher the sintering temperature, the denser the resulting part), but for 316L, higher temperatures promote a higher level of the δ-ferrite phase. Relative porosity may range between 1.7–5.6%, with the lowest values obtained under vacuum sintering [32]. Even lower values (1.08%) are reported in [31] for an optimized debinding and vacuum sintering and in [79], in which case the use of Hot Isostatic Pressing allowed reaching a fully dense condition (i.e., porosity < 0.1 %). According to the literature, the pores in the samples can be isolated spherical pores with a few irregular interconnected pores [32] or elongated pores with an elliptical shape, having their major axis aligned perpendicular to the build direction [79]. Unlike PBF and DED, the BJ process does not intrinsically favor (or aid) microstructural anisotropy, and the equiaxed grain structure is typically observed [56]. In contrast to PBF and DED, in which rapid melting and solidification cause the presence of a strong texture with elongated columnar grains in the build direction, grain growth, which takes place because of the high temperature and long dwelling time inside the furnace, does not lead to the formation of a growth-dependent texture. 

**Figure 11:**
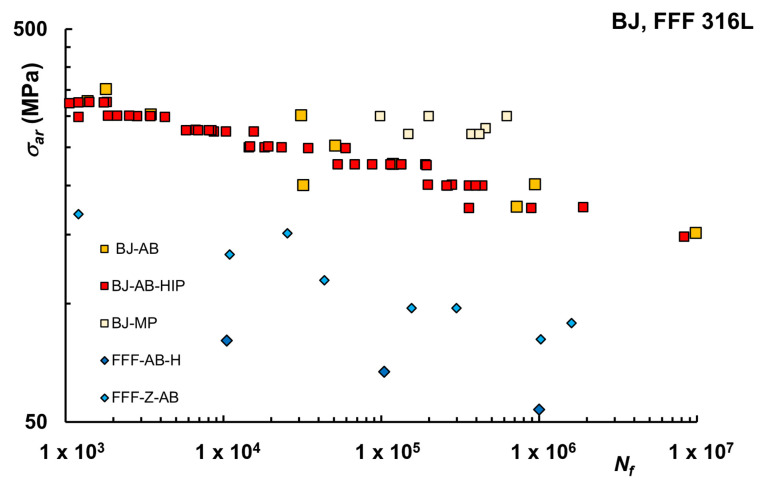
Fatigue life curves BJ and FFF 316L [36,56,66,78].

For FFF technologies, the microstructure was investigated in [15], and X-ray Diffraction (XRD) data of the 316L sintered sample solely showed γ (FCC) austenitic phase with full dissipation of the subtle presence of δ-ferrite detected in the powder feedstock. In [35], the nanoindentation technique revealed instead the presence of austenite, δ-ferrites as well as detrimental oxides and σ-phase depending on sintering conditions. The presence of the second phase (Cr-C) along the grain boundaries was also noticed in [36]. In addition, for FFF, a weakly textured almost random distribution of austenitic grains was reported in [15], with larger grains in the sintered sample than the wrought, whereas in [36], equiaxed grains were observed with size 40.5 ± μm. For FFF parts in [104], after parameter optimization throughout the entire SDS process, including printing and debinding, a final density above 95% of the theoretical density was reached. In [105], the highest relative density obtained was 92.9%, with little influence of layer orientation, whereas in [35], an average sintering density above 96% was reported. Overall the porosity of FFF is still higher than PBF and DED, in which case full densification can be achieved. Additionally, in this case fatigue data are limited and life curves for BJ 316L and FFF 316L are reported in Figure 11 [36,56,66,78].

According to [78], in which effects of the HIP on fatigue of as sintered specimens were investigated, BJ parts have fatigue properties similar to machined parts, despite the presence of abundant cracking due to high surface roughness. HIP did not affect the average fatigue strength but improved the cycle-to-failure consistency of sintered BJ parts. Unfortunately, porosity level and surface roughness were not reported, although polishing was suggested to improve fatigue strength by reducing the high surface roughness. Even higher fatigue strength is reported in [56] for machined and polished samples, showing that the higher porosity in the BJ specimens lowers YS but does not adversely affect HCF resistance, differently than SLM, in which case porosity has an adverse effect. This was attributed to a combination of the mechanisms that govern the first stage of the plastic deformation in the BJ alloys, coupled with microstructural features such as high-angle grain boundaries, δ-ferrite phase, and annealing twin boundaries. These make it difficult for the fatigue cracks to grow because they get arrested when they reach these microstructural obstacles, provided the applied stress amplitude is below the fatigue strength. Considering FFF, only two studies investigated fatigue of 316L [36,66] and even if the number of test data is indeed limited, as apparent from Figure 11, the fatigue behavior seems significantly lower than the other processes. According to [66], findings indicate that gaps at the connections between filaments lines are a cause of inferior mechanical properties, especially evident in the printed orientation Z, and further development of the metal FFF technology, also to improve strength and repeatability, is required before considering FFF parts for structural applications. Results presented in [36], where horizontally printed samples were tested, are even worse in terms of fatigue strength. 

## 7. Other Investigations on Fatigue of AM 316L

The literature discussed in the previous paragraphs was essentially focused on the application of Stress-based approaches to investigate the influence of processing or post-processing routes on the fatigue behavior of AM 316L. As observed, fatigue is a complex phenomenon that can be investigated with different aims or following different approaches.

### 7.1. Strain-Based Approaches and LCF

An alternative to the Stress-based approach is represented by the Strain-based approach, in which case strain is the control variable during the test. Traditionally this approach is used for investigations on the LCF regime, but it has been proven accurate even for higher cycle numbers. While for other AM metal alloys it is not uncommon to find strain-life curves, for 316L the number of studies is limited. In particular, for L-PBF it was adopted in [75,76], to investigate the effects of build orientation and surface roughness. These investigations showed that the horizontally built specimens possessed the highest fatigue resistance, while the least fatigue strength was obtained for diagonally built specimens, with defects resulting from a lack of fusion between the subsequent layers acting as the primary factors for failures. The effects of surface roughness (machined and polished samples) on initiating fatigue cracks were instead minor. In [77] LCF was instead considered in relation to HT parameters, determining coefficients of the Manson-Coffin equation for different conditions. Finally, a Strain-based approach was used in [79] for BJ 316L, testing machined specimens built horizontally, with or without HIP. LCF was investigated, showing that typical defects of BJ parts, like distributed fine residual pores, detrimental sigma phases, and carbides did not contribute to fatigue failure of the material. HIP reduced the scatter of the data but did not improve the average fatigue strength.

### 7.2. Fatigue Crack Propagation (FCP)

For AM metals the grain structure resulting from processing may affect fatigue crack propagation. As discussed in [8], in L-PBF-produced samples grain structure is composed of many solidification cells, elongated in the same direction as the grains, resulting in different crack propagation routes depending on the loading direction. When a load is applied parallel to the direction of grain growth, the crack path is more tortuous, resulting in slower growth. When a load is applied perpendicularly to the grain long axis, the crack propagation along grain boundaries is straight and unhindered. Investigations on FCP for AM 316L are currently limited. In [22] the fatigue crack growth characteristics of the SLM alloys were similar to those of conventionally manufactured alloys. In [69] it was reported that thanks to high ductility, crack growth behavior for SLM 316L is not significantly influenced by process-induced imperfections, i.e., pores and internal stresses. However, the fatigue crack growth behavior was drastically affected by solidification and resulting microstructure, consisting of columnar grains preferentially oriented in the building direction. Such anisotropy, typical of AB or SR conditions, results in different fatigue crack growth rates depending on the relation of crack growth direction and grain long axis. HIP may induce equiaxed grains, reducing these differences. The anisotropic behavior of SLM-manufactured materials was also confirmed in [73], where fatigue crack propagation was studied as a function of build orientation. In this case, heat treatments at 1050 °C did not change the elongated grain structure resulting from printing. However, as reported in [17], for L-PBF 316L the differences between FCP behavior in terms of the threshold value of stress intensity factor (ΔK_th_) or coefficients of Paris’ law of the vertical and horizontal specimens were limited.

### 7.3. Short Time Procedures

Fatigue testing campaigns can be highly time-consuming and expensive, especially when dealing with AM metal alloys, and many researchers have proposed methods for fast assessment of fatigue properties using a reduced number of specimens. In particular, for 316L in [70,72], a step method was adopted to estimate the sensitivity to internal and surface defects of fatigue endurance whereas in [71] fatigue limit evaluation based on thermographic methodology was used to compare the fatigue properties of SLM and rolled 316 steel parts, using the self-heating approach. In [53] the influence of SR was instead investigated by considering different short-time procedures to reduce the high material and time effort in fatigue investigations. In particular, the method PhyBaLCHT, based on cyclic indentation tests, was applied for the characterization of the materials’ defect tolerance, and load increase tests (LITs) were used for qualitative analyses of the cyclic deformation behavior, showing a good correlation with more traditional constant amplitude testing. 

### 7.4. Predictive Models

Advanced fatigue models were recently applied to AM 316L. In [71], a computational model based on crystal plasticity and mesoscopic fatigue criterion was developed to simulate the effect of grain size and ductility, on the fatigue limit. In [106], results of fatigue tests after various controlled changes in laser power and scan speed were studied by employing the statistical response surface method, developing a predictive model for the fatigue stress-life relations capable to account for the different failure mechanisms identified for low and high-cycle fatigue. In [52,107] adaptive neuro-fuzzy inference system was instead applied for fatigue life prediction of laser powder bed fusion (L-PBF) stainless steel 316L, providing a first example of the key role machine learning approaches may have in the future. Finally, in [74] the fatigue estimation of S-N curves was based on the dynamic multiswarm particle swarm optimizer (DMS-PSO) algorithm for parameter optimization of a three-parameter Weibull distribution model, with good consistency between the model and the metallographic and fractographic phenomena.

### 7.5. Critical Defect Size and Role of Porosity

Another relevant aspect investigated is the influence of diverse types of defects on fatigue. As discussed in [7] for AM metals primarily two types of porosity are usually considered, gas porosity from encapsulated gas due to incomplete melting of particles with internal porosity, and lack of fusion (LOF) due to voids that create among unmelted or incompletely melted powder particles. While defect characterization or porosity analyses are common in fatigue studies, a few works specifically focused on this aspect. In [72], deterministic defects were created inside the material to investigate their role in relation to fatigue resistance, using a Kitagawa diagram. This study showed that the dimensions at which a defect can become critical in fatigue depend mostly on its position, with surface pores being far more deleterious than internal ones.

Recently, investigation on the role of artificial defects was extended to VHCF [39], reporting in this case that the embedded artificial defects did not affect the VHCF behavior and crack initiation started from natural defects closer to the surface. In [60], the applicability of the √area-parameter model was evaluated, showing a clear trend that suggests that the model can describe the fatigue strength for defects with √area ≥ 1000 µm in L-PBF processed 316L steel. In [108] the competing influence of porosity and microstructure on the fatigue property of L-PBF 316L was instead investigated, using the Kitagawa diagram to estimate critical pore size. Different fatigue failure modes were reported, with grain boundary defect-driven crack initiation or porosity-driven depending on pore size. In [63] the role of microscopic defects on multiaxial fatigue lifetime of SLM 316L was investigated, taking advantage of XCT analyses. Finally, it should be noted that for other types of AM structural steels, the effects of fatigue loading on metallic structure, lifetime, and fracture surfaces have been investigated using microtomography and topographic measurements of the fracture surfaces [109]. These measurements not only showed quantitatively a relationship between fracture surface roughness and fatigue load level or type [110] but also that a reduction of the applied stress level resulted in a decrease in porosity after mechanical loading. For AM316L this approach is not yet documented but may represent an interesting development.

## 8. Conclusions

In the present review, the current literature on fatigue of AM 316L has been examined considering different AM technologies (i.e., PBF, DED, BJ, and FFF), in comparison with conventional processing routes, and main process-related factors. The available literature is mostly focused on the L-PBF process, with a prevalence of studies using a Stress-based approach. For the other technologies, the current literature is instead limited to a few preliminary investigations. In this scenario, the following conclusions are possible:

- Considering fatigue of L-PBF 316L, a distinct response is observed depending on whether AB or M/P condition is considered. For AB parts, run-out at 10^7^ cycles was reported for σ_ar_ up to around 130 MPa versus 330 MPa for wrought material (machined). After M/P operation, the fatigue strength of AM materials is closer (run-out at 10^7^ cycles for σ_ar_ up to around 220 MPa) to conventional parts, but still lower when considering parts printed vertically. Thus, available data show that some improvement for current technologies is still needed to make as-fabricated AM 316L competitive with its traditional counterparts. Surface treatments may improve fatigue response, but available data are limited, and more investigations are needed.
- Fatigue strength is sensitive to build orientation and for L-PBF 316L literature data confirm that samples printed flat are generally more resistant against fatigue. After machining, XY samples may reach run-out at 10^7^ cycles for σ_ar_ up to around 330 MPa, with a fatigue strength comparable with conventional 316L.
- Common HTs for 316L include SR, ANN, and HIP. For SR a more noticeable positive effect is observed for specimens with a surface in AB condition, whereas after machining the effect is limited. This supports the conclusion that relief of internal tensions is less effective on fatigue when surface layers have already been removed by some machining operation. ANN and HIP do not seem to provide distinct advantages for AB condition, while for machined conditions run-out at 10^7^ cycles for σ_ar_ up to around 300 MPa were reported. Some differences can also be noticed depending on whether low or high cyclic stress is of interest.
- Considering fatigue of L-DED 316L, results seem promising, with run-out at 10^7^ cycles for σ_ar_ up to around 210 MPa but current knowledge at the microstructural level is still limited. For BJ 316L, despite higher porosity levels, fatigue strength seems comparable with L-PBF under AB condition (run-out at 10^7^ cycles for σ_ar_ up to around 150 MPa), thanks to a peculiar fatigue failure mechanism. FFF 316L exhibited the lowest fatigue strength, as a consequence of higher porosity and lower internal cohesion between layers and fused filaments, requiring optimization of processing conditions.
- Taken as a whole, available fatigue data are highly scattered, and even if some general trends emerge, it is clear that a common and shared base for optimal selection of processing and post-processing parameters is still lacking, even for more mature processes like L-PBF. This is a consequence of the high number of processing parameters involved, combined with the availability on the market of different commercial systems that may require different settings or whose parameters selection is not disclosed or available for the end user.
- The microstructure of AM 316L is different from conventional manufacturing and plays a major role in fatigue crack initiation and propagation. For L-PBF the microstructure of 316L is generally fully austenitic with elongated grains, resulting in anisotropic resistance to crack propagation, with more tortuous paths and slower growth when the load is applied parallel to the direction of grain growth. Fine cellular microstructures, typical of AB condition, are deemed to be more favorable for higher stress levels. Annealing or HIP at high temperatures causing recrystallization may lead to more isotropic crack propagation and coarser-grained microstructure that can be preferred when higher ductility and lower cyclic stresses are needed.
- For other AM technologies less information is available, but studies indicate that in DED, the microstructure consists of both columnar and equiaxed grains, with δ-ferritic phase up to 4–5% and oxides or non-metallic inclusions that may favor crack initiation. In BJ, the δ-ferritic phase may also be present, but the microstructure is not elongated in the building direction and microstructural features such as high-angle grain boundaries and annealing twin boundaries may positively affect fatigue behavior, by making it more difficult for the fatigue cracks to grow. In FFF the higher porosity hinders other microstructure-related effects.
- While fatigue is an inherently complex phenomenon, especially for AM metal in which internal defects and surface roughness may play a crucial role, the variability observed for fatigue data may also be the consequence of the different testing protocols adopted, in which load ratio R, shape (flat or cylindrical) and size of the specimens may change considerably. A shared standardized approach among researchers would certainly be beneficial to better isolate contributions from process-related factors.

## Figures and Tables

**Table 1:** List of papers on fatigue of L-PBF 316L–Stress-based approach.

Ref.	Build Orientation	Surface Finish	Heat Treatment	Specimen		Test Type	R
[40]	Z	AB/M	N	C	HG	AX	0.1
[41]	Z	AB/M	N/SR/HIP	C	HG	AX	−1
[42]	XY/Z	AB	SR/HIP	C	HG	AX	−1
[43]	Z	AB/M/ST	N	C	DB	AX	−1
[1]	XY	M	N	F	DB	AX	0.1
[44]	XY	M	N/ANN	F	DB	AX	0.1
[45]	XY	M	N/ANN	F	DB	AX	0.1
[46]	XY/Z	M	N	C	DB	AX	−1
[47]	XY/Z/45°	M	N	C	DB	AX	−1
[48]	Z	AB/M	N/SR	C	HG	AX	−1
[49]	Z	AB	ANN	C	HG/DB	AX/RB	−1
[50]	Z	AB	N	C	DB	AX	0.1
[51]	XY/Z	AB/M/ST	N/SR	C	HG	AX	0.1
[52]	XY/Z	M	N/ANN/HIP	F	DB	AX	0.1/0.7/−1
[53]	XY/Z	M	SR	C	DB	AX	−1
[54]	Z	M	ANN, HIP	C	HG	AX	−1
[55]	Z	AB/M	N	C	DB	AX	0.1
[56]	Z	M	SR	C	HG	RB	−1
[57]	XY/Z	AB/M/ST	N	C	DB	AX	0.1
[58]	Z	AB/M	SR	C	HG	AX	0/−1
[59]	XY	N/D	N	F	DB	AX	0.1
[60]	XY	M	N	C	HG	AX	−1
[61]	Z	M	ANN	C	HG	AX	−1
[62]	XY	M	SR	C	DB	M-AX	N/A
[63]	XY	M	N/D	C	DB	M-AX	N/A
[64]	Z	M	ANN	C	DB	AX	−1
[65]	Z	M	ANN	C	DB	AX	−1
[66]	Z	AB	SR	F	DB	AX	0.1
[67]	Z	M/ST	SR	C	HG	RevB	−1
[68]	XY	AB/ST	SR	F	-	RevB	−1
[69]	XY/Z	-	SR	F	CT	FCP	0.1
[70]	Z	M	N	C	DB	AX/STP	0.1
[71]	-	M	N	C	HG	AX/STP	0.1
[72]	Z	M	SR	C	DB	AX/STP/AD	0.1
[73]	Z/45°	-	SR/ANN	F	CT	FCP	0.1
[74]	Z	M	N/HIP	F	DB	AX	0
[38]	Z	M	N	C	DB/HG	AX	0.1
[39]	Z	M	N	C	HG	AX/AD	0.1

**Table 2:** List of papers on fatigue of L-PBF 316L–Strain-based approach.

Ref.	Build Orientation	Surface Finish	Heat Treatment	Specimen		Test Type	R
[75]	XY/Z/45°	M	N	C	DB	AX	−1
[76]	XY/Z/45°	AB/M	N	C	DB	AX	−1
[77]	Z	AB	PH/HIP	C	DB	AX	0.1

**Table 3:** List of papers on fatigue of DED, BJ, FFF 316L.

Ref.	Process Technology	Build Orientation	Surface Finish	Heat Treatment	Specimen		Test Type	R
[46]	L-DED	XY/Z	M	N	C	DB	AX	−1
[78]	BJ	Z	AB	HIP	C	HG	AX	−1
[56]	BJ	Z	M	N	C	HG	RB	−1
[36]	FFF	XY	-	N	F	DB	RevB	0.1
[66]	FFF	Z	AB	N	F	DB	AX	0.1
[79]	BJ	XY	M	HIP	C	DB	AX	−1

## Data Availability

Not applicable.

## Nomenclature

AB	As-Built	ME	Metal Extrusion
AD	Artificial Defects	N	No Heat Treatment
AM	Additive Manufacturing	N_f_	Number of cycles to failure
ANN	Annealing	P	Polished
AX	Axial	PBF	Powder Bed Fusion
BJ	Binder Jetting	P_d_	Total Laser Energy in unit time
C	Cylindrical specimen	PH	Precipitation Hardening
CC	Continuous Casting		
CT	Compact Tension specimen	PhyBaL_CHT_	Short-time procedure based on cyclic indentation test
DB	Dogbone specimen	R	Fatigue Load Ratio
DED	Directed Energy Deposition	R_a_	Surface roughness Ra
Δ K_th_	Threshold value of stress intensity factor	RB	Rotating Bending
DMLS	Direct Metal Laser Sintering	RevB	Reversed Bending
DMS-PSO	Dynamic multiswarm particle swarm optimizer algorithm	R_z_	Surface roughness Rz
δ-phase	Ferritic phase	SR	Stress Relief
E	Elastic Modulus	ST	Surface Treatment
EBSD	Electron Backscatter Diffraction	SS	Stainless Steel
E_d_	Laser Energy Density	σ_a_	Alternating stress in a fatigue cycle
ε_R_	Strain at failure	σ_ar_	Equivalent alternating stress
F	Flat specimen	SLM	Selective Laser Melting
FCI	Fatigue Crack Initiation	σ_m_	Mean stress in a fatigue cycle
FCP	Fatigue Crack Propagation	σ_max_	Maximum stress in a fatigue cycle
FFF	Fused Filament Fabrication	σ_min_	Minimum stress in a fatigue cycle
γ-phase	Austenitic phase	σ-phase	Chromium/molybdenum-rich intermetallic phase
h	hatch spacing	SP	Shot Peening
HAGB	High Angle Grain Boundaries	STP	Stepped Test Protocol
HCF	High Cycle Fatigue	SWT	Smith-Watson-Topper parameter
HFMI	High-Frequency Mechanical Impact finishing	t	Layer Thickness
HG	Hourglass specimen	T	Torsional load
HIP	Hot Isostatic Pressing	UTS	Ultimate Tensile Strength
HT	Heat Treatment	v	Scanning speed
LCF	Low Cycle Fatigue	VF	Vibratory Finishing
L-PBF	Laser Powder Bed Fusion	VHCF	Very High Cycle Fatigue
L-DED-P	Directed Laser Deposition (Powder form)	WAAM	Wire Arc Additive Manufacturing
L-DED-W	Directed Laser Deposition (Wire form)	XCT	X-ray Computed Tomography
LENS	Laser engineered net shaping	XRD	X-ray Diffraction
LIT	Load Increase Tests	XY	Horizontal Building Orientation
LOF	Lack of Fusion	YS	Yield Strength
LSP	Laser Shot Peening	Z	Vertical Building Orientation
M	Machined	2N_f_	Number of reversals to failure
M-AX	Multiaxial load	45°	Diagonal Building Orientation
